# Evidence for treatable inborn errors of metabolism in a cohort of 187 Greek patients with autism spectrum disorder (ASD)

**DOI:** 10.3389/fnhum.2013.00858

**Published:** 2013-12-24

**Authors:** Martha Spilioti, Athanasios E. Evangeliou, Despoina Tramma, Zoe Theodoridou, Spyridon Metaxas, Eleni Michailidi, Eleni Bonti, Helen Frysira, A. Haidopoulou, Despoina Asprangathou, Aggelos J. Tsalkidis, Panagiotis Kardaras, Ron A. Wevers, Cornelis Jakobs, K. Michael Gibson

**Affiliations:** ^1^First Department of Neurology, AHEPA Hospital, Aristotle University of ThessalonikiThessaloniki, Greece; ^2^Fourth Department of Pediatrics, Papageorgiou Hospital, Aristotle University of ThessalonikiThessaloniki, Greece; ^3^Department of Special Educational Needs, St. Luke's HospitalThessaloniki, Greece; ^4^Second ENT Department, Papageorgiou Hospital, Aristotle University of ThessalonikiThessaloniki, Greece; ^5^Department of Pediatrics, Medical School, University of CreteHeraklion, Greece; ^6^Department of Pediatrics, Papageorgiou Hospital, Aristotle University of ThessalonikiThessaloniki, Greece; ^7^Department of Pediatrics, Athens University Medical School, Agia Sophia Children's HospitalAthens, Greece; ^8^Department of Pediatrics, Medical School, University of ThraceAlexandroupolis, Greece; ^9^Third Department of Pediatrics, Hippokration Hospital, Aristotle University of ThessalonikiThessaloniki, Greece; ^10^Laboratory of Genetic, Endocrine and Metabolic Diseases, Department of Laboratory Medicine, RUNMCNijmegen, Netherlands; ^11^Metabolic Unit, Department of Clinical Chemistry, VU University Medical CenterAmsterdam, Netherlands; ^12^Section of Clinical Pharmacology, College of Pharmacy, Washington State UniversitySpokane, WA, USA

**Keywords:** autism, inborn errors of metabolism, biotin, ketogenic diet, 3-hydroxyisovaleric acid, Lesch-Nyhan disease, succinic semialdehyde dehydrogenase deficiency, phenylketonuria

## Abstract

We screened for the presence of inborn errors of metabolism (IEM) in 187 children (105 males; 82 females, ages 4–14 years old) who presented with confirmed features of autism spectrum disorder (ASD). Twelve patients (7%) manifested increased 3-hydroxyisovaleric acid (3-OH-IVA) excretion in urine, and minor to significant improvement in autistic features was observed in seven patients following supplementation with biotin. Five diagnoses included: Lesch Nyhan syndrome (2), succinic semialdehyde dehydrogenase (SSADH) deficiency (2), and phenylketonuria (1) (2.7%). Additional metabolic disturbances suggestive of IEMs included two patients whose increased urine 3-OH-IVA was accompanied by elevated methylcitrate and lactate in sera, and 30 patients that showed abnormal glucose-loading tests. In the latter group, 16/30 patients manifested increased sera beta hydroxybutyrate (b-OH-b) production and 18/30 had a paradoxical increase of sera lactate. Six patients with elevated b-OH-b in sera showed improved autistic features following implementation of a ketogenic diet (KD). Five patients showed decreased serum ketone body production with glucose loading. Twelve of 187 patients demonstrated non-specific MRI pathology, while 25/187 had abnormal electroencephalogram (EEG) findings. Finally, family history was positive for 22/187 patients (1st or 2nd degree relative with comparable symptomatology) and consanguinity was documented for 12/187 patients. Our data provide evidence for a new biomarker (3-OH-IVA) and novel treatment approaches in ASD patients. Concise 1 sentence take-home message: Detailed metabolic screening in a Greek cohort of ASD patients revealed biomarkers (urine 3-hydroxyisovaleric acid and serum b-OH-b) in 7% (13/187) of patients for whom biotin supplementation or institution of a KD resulted in mild to significant clinical improvement in autistic features.

## Introduction

Numerous pathologies (Fragile X, syndromes of congenital infection, vaccinations, perinatal damage, and others) have been discussed as potential etiologies associated with autistic behavior and/or autism spectrum disorder (ASD) (Mazzoko et al., 1998; Gallagher and Goodman, 2010), and an expanding literature has demonstrated more frequent associations between inborn errors of metabolisms (IEMs) and ASD (Weissman et al., 2008; Sempere et al., 2010). Recent reports have highlighted a growing association between ASDs and respiratory chain abnormalities, including complex III/IV deficiency and MELAS syndrome, as well as glucose-6-phosphate dehydrogenase deficiency (Guevara-Campos et al., 2010; Chauhan et al., 2011). Further, a number of reports suggest metabolic derangements in ASD patients that are suggestive of IEM, such as abnormalities of glucose oxidation and utilization, among others (Haznedar et al., 2006; Zhao et al., 2010). In these cases, however, it is difficult to conclusively identify whether the manifestation of ASD on the background of the IEM is a primary or secondary pathology. Despite the increased number of IEM, recent work (Schiff et al., 2010) suggested that a careful clinical evaluation is more crucial than systematic metabolic investigations but this aspect should be tested through a large population based prospective study assessing the benefits of routine metabolic screening in non-syndromic autistic spectrum disorders.

Greece represents a country for which a number of populations reside in relative geographic isolation. This is especially true for the Greek islands, where previous studies have documented a large number of patients for whom indeterminate neurological features associate with a small number of IEMs (Evangeliou et al., 2001). Many of these patients manifest features of ASD, occasionally associated with a positive family history and/or consanguinity. In order to extend our earlier work, we examined a cohort of these patients on the island of Crete in order to ascertain the incidence of IEMs and metabolic disturbances, and the corresponding link to ASD. The current report summarizes the results of our investigations.

## Materials and methods

We evaluated 187 children (105 males, 82 females; ages 4–14 years) who presented with ASD. The differential diagnosis was based upon DSM-IV criteria for pervasive developmental disorders (PDDs; Kim et al., 2010; see also Table 1).

**Table 1:** **Childhood Autism Rating Scale (CARS) (Schopler et al., 1980)**.

1.	Disorder in human relationship (i.e., no appreciation by the individual of the interest that other people show for him/her)
2.	Mimicking (i.e., the extent to which the patient mimics)
3.	Improper emotions (e.g., the unsuitable timing of emotions such as laughing and crying)
4.	Bizarre use of bodily movements and persistence to stereotypy
5.	Peculiar relations with objects (e.g., correct use of objects)
6.	Resistance to changes in the environment
7.	Idiosyncratic optic reactions (e.g., avoidance of eye contact)
8.	Idiosyncratic acoustic reactions (avoidance of or exaggerated reaction to noise)
9.	Putting objects in mouth, licking, smelling, and rubbing
10.	Stress reactions (e.g., intensity of repression)
11.	Verbal communication (e.g., lack of speech, echolalia, replacement of personal pronouns)
12.	Nonverbal communication (e.g., use of or response to gestures)
13.	Extreme levels of activity (e.g., apathy or hyperactivity)
14.	Mental function (i.e., lack of homogeneity of cognitive characteristics)
15.	General impressions (e.g., general ranking)

Legend to table: ranking of symptoms: 1, normal for age; 2, mild disorder; 3, moderate disorder; 4, serious disorder. For each of these 15 items a cumulative score is derived by summing 1–4 points for each item.

Patients with identified ASD etiologies (e.g., perinatal damage, CNS infection, CNS tumor, or chromosomal abnormalities related to known neurogenetic disorders such as Angelman syndrome) were excluded from evaluation. Further, patients with otorhinal or ocular abnormalities, as well as failure to thrive, were also excluded.

At admission, we recorded detailed family histories (e.g., additional affected individuals, consanguinity, repetitive miscarriages, etc.) as well as dietary habits of the patients (symptoms following ingestion of certain foods and/or prolonged fasting, tendency to dietary avoidances). All subjects underwent detailed clinical and psychiatric examination, and anthropomorphic data was obtained. Laboratory investigations included: complete blood count, blood biochemical evaluations (electrolytes, glucose, transaminases, CPK, LDH, cholesterol, triglycerides, thyroid hormones), electrocardiogram (ECG), and electroencephalogram (EEG). These were followed by more specific examinations, including: serum amino acids, carnitine, urine purines and pyrimidines, urine amino and organic acids, urine mucopolysaccharides and oligosaccharides, cytogenetic analysis, and glucose loading test. For the latter, blood was obtained for determination of lactic acid, pyruvate, and β-hydroxybutyric acid (b-OH-b) following an 8 h fast, after which subjects received a 10% glucose bolus (2 g/kg of body weight, maximum 50 g). Sixty minutes post glucose administration, blood was obtained for determination of the same metabolites. In selected patients, more detailed analyses included lysosomal enzymology, guanidinoacetate-*n*-methyltransferase (GAMT) assay and biopsy of skeletal muscle for assessment of various mitochondrial enzymes.

## Results

### Confirmed diagnoses

We identified two patients with Lesch Nyhan syndrome, two patients with succinic semialdehyde dehydrogenase (SSADH) deficiency, and a single patient with phenylketonuria. Thus, confirmed diagnoses in ASD patients for a known IEM was 2.7% of the total subject number investigated.

### Biochemical abnormalities suggestive of IEM

For 12/187 (7%) of patients, urinary 3-hydroxyisovaleric acid (3-OH-IVA) was elevated and sera methylcitrate and lactate levels were also elevated in two of these patients. Despite these biochemical abnormalities, defects in biotinidase, or holocarboxylase synthetase (Watanabe et al., 2005) could not be demonstrated in either sera or fibroblasts. Of interest, none of these 12 patients was undergoing valproate intervention, the latter a potential source of 3-OH-IVA elevation in urine (Silva et al., 2001). Despite an absence of confirmatory enzyme deficiencies in these 12 patients, we nonetheless opted to treat empirically with biotin for 3 weeks, 2 × 10 mg and then for 6 months at 2 × 5 mg, which led to a clear therapeutic benefit in 7/13 consisting of improvement in the Childhood Autism Rating Scale (CARS; Table 2). For those benefiting from biotin intervention, the most impressive outcome centered on a 42 month-old boy whose severe ASD was completely ameliorated following biotin intervention. This patient was subsequently followed for 5 years, and cessation of biotin intervention (or placebo replacement) resulted in the rapid return of ASD-like symptomatology. This patient currently attends public school without any clinical sequelae and remains on biotin at 20 mg/d.

**Table 2:** **ASD patients with increased urinary 3-hydroxyisovaleric acid and biotin supplemented**.

**Pat. no**	**3-OH-IVA mmol/mol/creatinine (normal range 10.4–67.0)**	**Cardinal symptoms**	**Non-autistic symptoms**	**Pre-CARS**	**Age of treatment initiation in years**	**Post-CARS 6 months after treatment initiation**	**Cardinal symptoms 6 months after treatment initiation**
1	171	H, AD, not responding to verbal cues, no speech, stereotypies, aggressive or self-injurious behavior, fascination with repetitive movement	No	44	3	19	No symptoms
2	161	H, AD, difficulty interacting with other people, compulsive behavior, speech abnormal in content and quality	Positive family history	44	3	29	Very mild H, very mild AD, amelioration of all other symptoms
3	200	No speech, H, AD, compulsive behavior, stereotypies	Consanguinity	41	4	29	Very mild H, very mild AD, amelioration of all other symptoms
4	115	Compulsive behavior, abnormal speech, H, AD, fascination with repetitive movement	No	41	4	32	Improvement in all symptoms
5	145	Speech abnormal in content and quality, H, AD	No	40	5	31	Improvement in all symptoms
6	145	Abnormal speech, H, AD	Pathologic EEG	38	2,5	30	Improvement in all symptoms
7	170	H, AD, Compulsive behavior, not responding to verbal cues, no speech, preoccupation with parts of objects, aggressive or self-injurious behavior	No	45	3	40	Still H and AD, some improvement in speech and behavior
8	182	Abnormal speech, H, AD, aggressive or self-injurious behavior	No	41	5	41	No improvement
9	175	Compulsive behavior, AD, abnormal speech, stereotypies	No	39	6	39	No improvement
10	128	Compulsive behavior, abnormal speech, H, AD, fascination with repetitive movement, stereotypies	No	41	4,5	41	No improvement
11	135	No speech, stereotypies, compulsive behavior, AD	No	41	4,4	41	No improvement
12	119	No speech, compulsive behavior, AD, aggressive or self-injurious behavior	No	38	5	38	No improvement

Legend to table: >12 unit difference is considered significant improvement; 8–12 unit, average improvement; 2–8 units, minor improvement. Note that a score of >30 on the CARS is indicative of autism. Patients were not undergoing adjuvant therapy during biotin intervention. Abbreviations: 3-OH-IVA, 3-hydroxyisovaleric acid; attention deficit, AD; hyperactivity, H.

### Results of diagnostic loading studies

A glucose challenge was administered to all test subjects, revealing a pathological increase in sera b-OH-b in 16/187 subjects (Table 3) and elevated sera lactate in 18/187. These results for glucose loading pointed to mitochondrial disease, although confirmatory testing could not be undertaken due to a lack of monetary support for muscle biopsy from both parents and insurers.

**Table 3:** **Evaluation of lactate and beta-hydroxybutyrate after glucose loading test**.

**Pat. no**	**Pre-GLT (b-OH-b)**	**Post-GLT (b-OH-b)**	**Pat. no**	**Pre-GLT (lactate)**	**Post-GLT (lactate)**
1	1.45	1.82	1	14	19
2	1.05	1.49	2	10	14
3	1.23	1.65	3	15	21
4	1.45	1.88	4	8	10
5	1.30	1.45	5	10	13
6	1.15	1.34	6	12	17
7	1.35	1.42	7	14	18
8	1.20	1.36	8	14	19
9	1.00	1.25	9	12	14
10	0.90	1.10	10	9	12
11	1.30	1.45	11	8	9
12	1.40	1.60	12	10	13
13	1.25	1.35	13	16	35
14	1.30	1.55	14	15	35
15	1.10	1.25	15	12	29
16	1.35	1.45	16	12	34

Patients with pathologically increased sera beta-hydroxybutyrate and lactate following glucose loading test (GLT). Patients 13–16 had both increased b-OH-b (beta-hydroxybutyrate) and lactate. Values for b-OH-b are in mmol/L, while those for lactate are in mg/dL. According to the kit employed for measurement of these metabolites, pathological values for b-OH-b pathological are considered >1.5 mmol/L, while pathological levels of lactate are considered as those >15 mg/dL. Pathological results for post-GLT lactate are those that exceed 30% of the pre-GLT lactate value.

**Figure 1:**
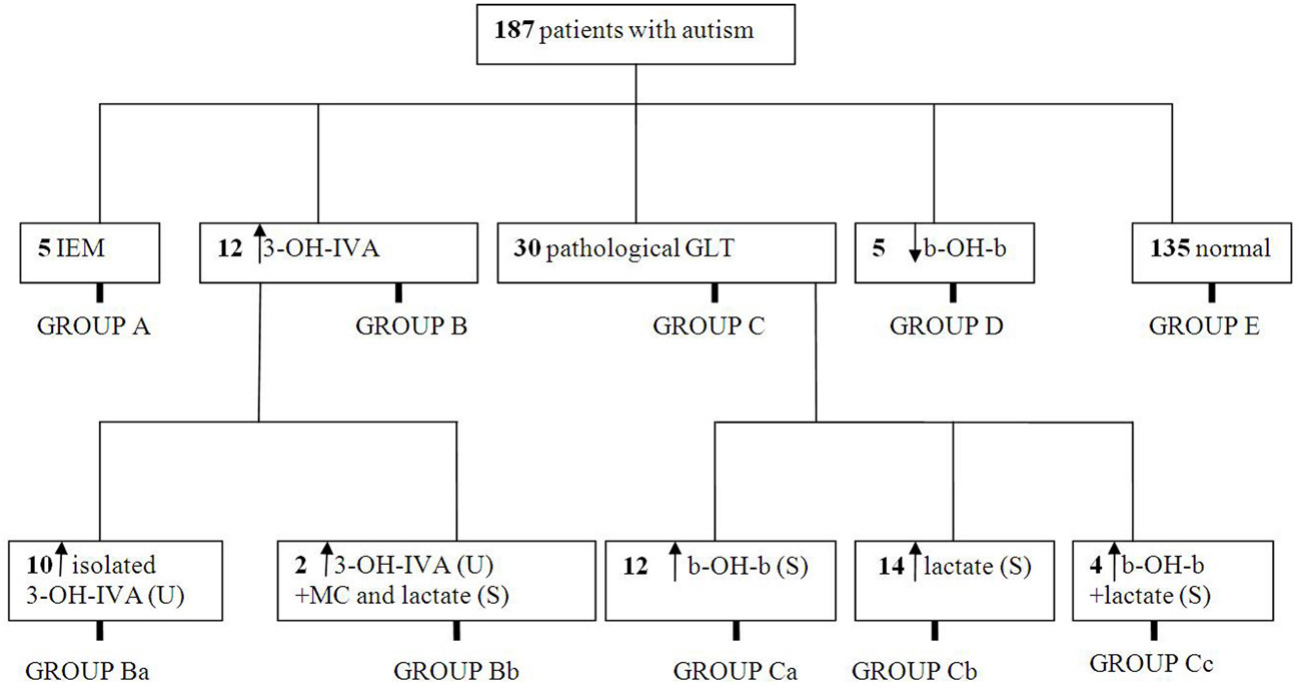
**Summary of metabolic abnormalities in the patients evaluated in this study**. Abbreviations: IEM, inborn errors of metabolism; 3-OH-IVA, 3 hydroxyisovaleric acid; GLT, glucose loading test; b-OH-b, β-hydroxybutyrate; MC, methylcitrate.

We instituted a ketogenic diet (KD) in 6/16 patients who had demonstrated a pathological increase in serum b-OH-b associated with glucose loading. Although our desire was to implement the KD in all 16 patients, it is a diet particularly challenging to implement in autistic patients, and we were only successful in six cases. One of these six patients showed a remarkable improvement in CARS scale (Table 4) and subsequently medications (risperidone and hydroxyzine) were stopped. This patient is currently attending a public elementary school without clinical problems. Clinical improvements in the remaining 5/6 patients were more subtle (Table 4). Additionally, for 5/178 patients, blood b-OH-b levels were significantly lower in comparison to control levels following a 12 h fast. Conversely, urine organic acid analysis and acylcarnitine evaluation in dried blood spots were normal for all of these patients, thus providing no evidence for a defect in beta-oxidation. An overview of abnormal biochemical and loading results is presented in Figure 1.

**Table 4:** **ASD patients responsive to ketogenic diet implementation**.

**Pat. no**	**Cardinal symptoms**	**Non-autistic symptoms**	**Pre-CARS**	**Age of treatment initiation in years**	**Post-CARS 6 months after treatment initiation**	**Cardinal symptoms 6 months after treatment initiation**
1	Hyperactivity, attention deficit, not responding to verbal cues, no speech, compulsive behavior, preoccupation with parts of objects, abnormal sleep	Pathologic EEG	41	3,5	21	No symptoms
2	Hyperactivity, attention deficit, not responding to verbal cues, no speech, aggressive or self-injurious behavior	Pathologic MRI	41	6	32	Improvement of all symptoms, no hyperactivity, no attention deficit
3	Hyperactivity, attention deficit, sustained inappropriate play, compulsive behavior, abnormal speech	Pathologic EEG	41	4	33	Improvement of all symptoms especially in speech, no hyperactivity
4	Hyperactivity, attention deficit, not responding to verbal cues, stereotype	Consanguinity	39	3,5	30	Improvement of all symptoms
5	Hyperactivity, attention deficit, not responding to verbal cues, no speech, compulsive behavior, preoccupation with parts of objects, sustained inappropriate play	Positive family history	45	4	40	Minimal improvement of all symptoms
6	Hyperactivity, attention deficit, no speech, compulsive behavior, sustained inappropriate play	Food intolerance (aggravation of symptoms after carbohydrate ingestion)	41	5	37	Moderate improvement in speech and attention deficit; minimal improvement of remaining symptoms

See legend to Table 2 for details. Patients were not undergoing adjuvant therapy during ketogenic diet intervention.

### Family histories, clinical findings, and dietary characteristics

Twenty-two of 178 test subjects had a family history that was positive for neurological disease. Additionally, 13/178 subjects had 1st, 2nd, or 3rd degree relatives with comparable symptoms, while other family members often suffered from other chronic neurological morbidity, including epilepsy, ataxia or mild to severe developmental delay. Consanguinity was confirmed in the parents of six patients (1st, 2nd and 3rd degree relatives). Unfortunately three of these families had children with symptomatology similar to that of the proband we investigated. Twelve of 178 patients had clear evidence of facial dysmorphia, including hypertelorism and low-set ears. With regard to diet, 26/178 subjects showed evidence of dietary intolerance. Of these, 5/26 patients (who also had abnormal glucose loading results) manifested exacerbation of symptoms during high carbohydrate intake. Similarly, three patients who demonstrated decreased blood ketone body production following a glucose bolus self-selected a low-fat diet, and high fat consumption correlated with deterioration in the clinical picture for one of these three, characterized by increased hyperactivity and stereotypies. Finally, 15/187 patients without biochemical abnormalities manifested food intolerance, and key clinical symptoms (hyperactivity, increased stereotypies, sleep disturbances) were exacerbated with high protein consumption.

### Neuroimaging abnormalities

Twenty-five of 187 subjects manifested pathological EEG findings without seizures, the most common feature being beta-rhythms. Abnormalities of the MRI were found in 12/187 subjects, featuring primarily cerebellar hypoplasia and agenesis of the corpus callosum in the absence of specific structural abnormalities. Additionally, 7/187 patients without pathological biochemical findings suffered from epilepsy that was treated symptomatically with valproate (*n* = 3), carbamazepine (*n* = 3) and oxcarbazepine (*n* = 1). A comprehensive summary of findings for family history, consanguinity, dysmorphia, imaging abnormalities and dietary aversions is presented in Table 5.

**Table 5:** **Distribution of pathological findings corresponding to subgroups depicted in Figure 1**.

	**Number of patients**	**Group A**	**Group B**		**Group C**			**Group D**	**Group E**
			**Ba**	**Bb**	**Ca**	**Cb**	**Cc**		
PathologicMRI	12			1	2	3	1		5
Pathologic EEG	28	2 (SSADH)	3	1	2	6	2	1	10
Dysmorphia	12				1	1	1		9
Food intolerance	27	1 (PKU)	2	1	3	2	2	4	13
Family history	22		2	1	2	6	1	1	9
Consanguinity	12		1		2	2			7
Diagnosed IEM	5	5							

The same patient can have more than one or no pathological findings.

## Discussion

A complex disorder associated with multifactorial inheritance, autism (or ASD; also pervasive developmental delay) is comprised of multiple phenotypic features, the most prominent of which are behavioral disturbances (e.g., obsessive compulsive disorder and/or highly ritualistic behavior) and primary disturbances of social skills, the latter prominent in adolescents and adults (Stokstad, 2001; Manzi et al., 2008; Kotulska and Jóźwiak, 2011). For most patients, the primary etiology remains undefined. Conversely, in a small subset of cases there is a clear genetic etiology, primarily those of a syndromic genetic disorder, including Fragile X syndrome, tuberous sclerosis and others (Pickler and Elias, 2009; Toriello, 2012). Additionally, expanding research has revealed the presence of ASD in IEM, including phenylketonuria, disorders of mitochondrial metabolism (Weissman et al., 2008; Shoffner et al., 2010), defects in the metabolism of purines and pyrimidines, and disorders of cerebral glucose transport (Schaefer and Lutz, 2006; Schiff et al., 2010; Zhao et al., 2010). Of interest, in the rare disorder of GABA metabolism, SSADH deficiency, a major subset of confirmed adolescent patients suffer from extensive obsession compulsion, frequently characterized as ASD (Pearl et al., 2011).

In addition to the confirmed cases of IEM that were detected in the current report (5/178 cases), our cohort analyses included indirect evidence for IEM without confirmed diagnosis, including abnormal responses to glucose loading, response to KD (in the absence of clinical seizures) and responsiveness to pharmacological biotin administration. The response to biotin in a subset of our cohort, despite an absence of defined deficiencies in biotin-dependent biotinidase or multiple carboxylase enzymes, supports earlier findings in ASD cohorts revealing nutritional deficiencies including biotin (Main et al., 2010; Adams et al., 2011). From the biochemical perspective, it would seem logical to assume that biotin response and hyperexcretion of 3-OH-IVA in our cohort are correlated, most likely through biotin-dependent 3-methylcrotonyl-CoA carboxylase, but currently we have no clear etiology explaining the response to biotin.

To our knowledge, the existence of Lesch-Nyhan disease or SSADH deficiency has not been previously detected during screening of any ASD patient cohort. The rarity of both suggests that a more discrete screening for these disorders in the ASD population is warranted, at the very least in the Greek population. The finding of two cases of SSADH deficiency within 187 cases (>1%) is consistent with the expanding phenotypic observation of OCD in these patients (Vogel et al., 2012). The current report, therefore, may present some justification for the concept of screening for SSADH deficiency in the autism and ASD populations, especially in those populations in which autosomal recessive disorders are expected to have increased prevalence.

The current report represents only the second large scale evaluation of ASD patients for the presence of IEMs. Schiff et al. (2011) broadly screened 274 ASD patients for the presence of IEM, identifying two cases. These included a case of non-specific urinary creatine excretion and a patient with persistent 3-methylglutaconic aciduria, or less than 1% yield in their patient cohort. Neither Schiff's report nor ours positively identified patients with mitochondrial disease, creatine transport defects, glucose transport defect or others (e.g., glucose-6-phosphate dehydrogenase deficiency) which have been previously documented in ASD patients (Connolly et al., 2010; Guevara-Campos et al., 2010). This may simply reflect the ethnicity of the cohorts evaluated, including France and Greece, where incidence/prevalence of various IEMs will be quite different. Other investigators have recommended that care be taken in considering screening for IEM in ASD patients (Wang et al., 2010), and Moss and Howlin (2009) have appropriately cautioned that correlation of behavioral similarities between ASD and those observed in genetically-determined syndromes (e.g., Fragile X, Angelman, etc.) be carefully interpreted. Nonetheless, our report suggests that further consideration be given to the selected analysis of IEM in ASD patients, and that those studies might benefit from the broadest coverage of ethnic and regional groups possible, especially in populations for whom recessive disorders have an increased incidence. Broad screening evaluations such as these might be justifiable in helping to identify genetic subsets of ASD, providing genetic counseling opportunities for affected families, and presenting treatment options for those disorders for which therapeutic options are available. Clearly, the key outcome of our investigation is the identification of biomarker (3-OH-IVA and b-OH-b) with therapeutic relevance (biotin, KD) for patients with ASD, suggesting that our results should be investigated in additional ASD cohorts.

### Conflict of interest statement

The authors declare that the research was conducted in the absence of any commercial or financial relationships that could be construed as a potential conflict of interest.
